# Effect of informatization-based blood glucose team management on the control of hyperglycaemia in noncritical care units

**DOI:** 10.1371/journal.pone.0230115

**Published:** 2020-03-11

**Authors:** Ying Zhu, Yan Yang, Miao Yang, Wei Xia, Hui Zhou, Xian-Jun Zhu, Nie Tang, Peng-Qiu Li

**Affiliations:** Department of Endocrinology, Sichuan Academy of Medical Sciences & Sichuan Provincial People’s Hospital, Chengdu, China; Chinese Academy of Medical Sciences and Peking Union Medical College, CHINA

## Abstract

**Purpose:**

To provide a new system of in-hospital blood glucose team management combined with a network blood glucose monitoring system and analyse the effect on hyperglycaemic participants’ blood glucose control in noncritical care units.

**Methods:**

Hyperglycaemic participants in noncritical care units were divided into two groups. They underwent active intervention by the hospital’s blood glucose management team or the routine consultation group. The better method, based on a shorter length of stay (LOS) and lower hospital cost, could be selected by comparing the two blood glucose management strategies.

**Results:**

Compared with the routine consultation group, the team management group had a higher detection rate of hyperglycaemia (18.49% vs 16.17%, P<0.01) and glycosylated haemoglobin (51.53% vs 30.97%, P<0.01) and a lower incidence rate of hyperglycaemia (59.24% vs 61.59%, P<0.01), severe hyperglycaemia (3.56% vs 5.19%, P<0.01) and clinically significant hypoglycaemia (0.26% vs 0.35%, P<0.05). Simultaneously, blood glucose drift (mmol/L) (2.50 (1.83, 3.25) vs 2.76 (2.01, 3.57), P<0.01), blood glucose coefficient of variation (%) (28.86 (22.70, 34.83) vs 29.80 (23.47, 36.13), P<0.01), maximum blood glucose fluctuation (mmol/L) (9.30 (6.20, 13.10) vs 10.10 (7.00, 14.40), P<0.01) and nosocomial infection (5.42% vs 8.05%, P<0.05) were all lower among participants in the team management group. In addition, the LOS (P<0.001) and hospital costs (P<0.001) of participants were lower in the team management group.

**Conclusion:**

In-hospital blood glucose team management combined with a network blood glucose monitoring system effectively improved the blood glucose control and fluctuation levels of participants who were admitted to noncritical care units, thereby reducing LOS and hospital cost.

## Introduction

In recent years, the prevalence of diabetes, especially among non-endocrinology patients, has increased in China due to the large changes in living standards and lifestyle [1]. Manually recorded blood glucose data and traditional consultation modes are still used to manage blood glucose in most hospitals. This method has many drawbacks, such as the inability to follow up people’s blood glucose, lack of involvement of diabetes education nurses, the lack relevant knowledge of non-endocrine specialists and the inability to pay sufficient attention to blood glucose. Therefore, it is often difficult for hospitalized people with hyperglycaemia to meet the desirable blood glucose standards [2]. Furthermore, managing diabetes serves as a long-standing investment for families, medical providers and even insurance companies. A previous study reported that diabetes mismanagement, including the management of hypoglycaemia and hyperglycaemia, was strongly related to increased hospital cost, morbidity and even mortality [3].

With the rapid development of information technology, the networking of various medical data, including blood glucose data, has been realized. Previous studies have mostly adapted a network model to manage blood glucose [4–9]. For example, a randomized controlled trial used a laptop-based predictive control algorithm to monitor the blood glucose level during perioperative and postoperative periods [4]. Lin *et al* used a computerized system that automatically identified people’s blood glucose values by using a Nova StatStrip Glucose Meter (glucose values ranging from 10 mg/dL to 600 mg/dL), which scanned and recorded an identification number from a bar code on the patient’s hand [5]. However, the use of computers combined with a professional blood sugar management team to manage blood glucose has never been reported.

In this study, the blood glucose of patients treated by the management team was managed by endocrinologists and nurse practitioners (NPs) in combination with a network blood glucose monitoring system. Compared with the routine group, the management group exhibited better blood glucose control, which could be concluded from the lower incidence rate of blood glucose drift, hyperglycaemia, severe hyperglycaemia, clinically significant hypoglycaemia and nosocomial infection. Moreover, the new hospitalized blood glucose team management strategy could reduce length of stay (LOS) and hospital cost. Our results suggest that team management combined with a network blood glucose monitoring system could serve as a promising method to manage blood glucose.

## Participants and methods

### Ethics approval

The study was performed in accordance with the Declaration of Helsinki and was approved by the Medical Ethics Committee of Sichuan Provincial People’s Hospital and Sichuan Academy of Medical Sciences (No. 2019–233). Written informed consent was obtained from all individual participants.

### Participants

A total of 4787 participants were admitted to the non-critical care units from July 1st to December 31st, 2018, and 885 of the patients with hyperglycaemia were included in the team management group and were treated with an in-hospital blood glucose management strategy with active intervention. A total of 4534 participants were admitted to the noncritical care units from July 1st to December 31st, 2017, and 733 of the patients with hyperglycaemia were included in the control group, and blood glucose was managed by following a routine consultation strategy. The inclusion criteria were as follows: 1) people with hyperglycaemia who had plasma glucose levels higher than 7.8 mmol/L at any time during hospitalization [2, 10] and 2) age >18 years; the exclusion criteria were as follows: 1) ICU transfer during hospitalization and 2) poor compliance.

### Protocols

The management team was composed of endocrinologists and education nurses. Blood glucose monitoring data uploaded by the Nova Blood Glucose Meter (Nova StatStrip 1.75, Johnson & Johnson, United States) were remotely read. After analysing the blood glucose monitoring data, the management team set the warning values for hyperglycaemia and hypoglycaemia and actively managed blood glucose for participants whose blood glucose levels did not meet the standards. Moreover, professional diabetes nurses met one-on-one with participants according to their specific conditions and followed up daily regarding the participant’s blood glucose management. The traditional consultation mode was adopted by the routine consultation group. Glucose data were recorded manually, and a non-endocrinologist sent a referral for endocrinology consultation if they thought a participant’s blood glucose was unsatisfactory. Subsequently, endocrinologists treated participants in line with the consultation referral and write their opinion but would not follow up regarding the implementation of the consultation or the blood glucose levels of participants.

### Outcome evaluation

The following indicators were retrospectively analysed in the two groups: basic indicators, indicators related to blood glucose management, blood glucose control indicators, blood glucose fluctuation index, and other effective management indicators. Basic indicators included age, sex, proportion of diagnosed diabetic participants among all participants, and average blood glucose 24 hours after admission. Management indicators related to blood glucose included the detection rate of hyperglycaemia and glycosylated haemoglobin, the testing frequency per person per day, and the proportion of participants who used insulin subcutaneously during hospitalization. Blood glucose control indicators included the average blood glucose level, the incidence of hyperglycaemia (blood glucose > 7.8 mmol/L), the incidence of critical hyperglycaemia (blood glucose > 16.7 mmol/L), the incidence of hypoglycaemia (blood glucose ≤ 3.9 mmol/L), the incidence of clinically significant hypoglycaemia (blood glucose <3.0 mmol/L), and the target blood glucose compliance rate (hierarchical management of participants and different glycaemic control goals in combination with the reasons for admission and the participants’ disease status were recorded) [2, 10] (Table 1). The blood glucose fluctuation index included the blood glucose drift (the standard deviation of blood glucose value, SD), the coefficient of variation of blood glucose (CV, blood glucose drift × 100%/average blood glucose), and the largest fluctuation range of blood glucose (LAGE, blood glucose maximum minus blood glucose minimum). Other effective management indicators included the proportion of participants with nosocomial infections, LOS, and hospitalization expenses. The impact of different management models on hospitalization days and hospitalization cost was explored by comparing these indicators.

**Table 1:** Glycemic control target stratification of inpatients.

	Strict	Ordinary	Loose
Empty or preprandial blood glucose (mmol/L)	4.40–6.10	6.10–7.80	7.80–10
2h postprandial or random blood glucose (mmol/L)	6.10–7.80	7.80–10	7.80–13.90

### Statistical analysis

The statistical analysis was performed using SPSS 22.0 software (SPSS Inc., Chicago, IL, USA). The χ^2^ test was used to compare the rates or ratios between the two groups. All non-normally distributed variables are presented as medians (median (P25, P75)) and IQRs. Differences between the groups were evaluated using the Mann-Whitney U rank sum test, as appropriate. The participant LOS and hospital cost were taken as dependent variables (LOS and hospital cost were all non-normally distributed, so they were converted to a normal distribution by logarithmic transformation). Multivariate linear regression analysis was used to identify influencing factors of the LOS and hospital cost. P < 0.05 was considered to indicate a significant difference.

## Results

### Basic characteristics of hospitalized patients

A total of 885 participants with hyperglycaemia were identified in the team management group. A total of 733 participants with hyperglycaemia were identified in the routine consultation group. There were no significant differences in age, sex, body mass index (BMI), proportion of diagnosed diabetic participants among the total participants, or average blood glucose 24 hours after admission (Table 2).

**Table 2:** Some index at admission.

	Team management group (n = 885)	Routine consultation group (n = 733)	*P* value
Basic index at admission			
Average age (M (P_25_, P_75_))	60 (48, 70)	61 (49, 71)	0.074
Male patients (%)	60.90	60.30	0.805
Female patients (%)	39.10	39.70	0.805
BMI (Kg/m2, M (P25, P75))	23.74 (20.54, 27.71)	25.11 (21.10, 29.05)	0.734
Diagnosed diabetes patients (%)	10.67	10.92	0.706
Average blood glucose after admission for 24 hours (mmol/L, M (P_25_, P_75_))	9.75 (7.40, 13.50)	9.30 (7.40, 13.60)	0.144
Management indicators about blood glucose			
Detection rate of hyperglycemia (%)	18.49	16.17	0.003
Glycated hemoglobin (%)	51.53	30.97	<0.001
Frequency of blood glucose testing per person per day (M (P_25_, P_75_))	3.67 (3.10, 4.33)	3.03 (2.62, 3.67)	<0.001
Subcutaneous insulin during admission (%)	36.61	18.96	<0.001
Indicators of blood glucose control			
Mean of glycemia (mmol/L, M (P_25_, P_75_))	8.50 (6.80, 11.00)	8.80 (6.90, 11.50)	<0.001
Incidence of hyperglycemia (%)	59.24	61.59	<0.001
Critical hyperglycemia (%)	3.56	5.19	<0.001
Hypoglycemia (%)	1.17	1.33	0.088
Clinically significant hypoglycemia (%)	0.26	0.35	0.035
Target blood glucose compliance rate (%)	65.21	61.82	<0.001
Index of blood glucose fluctuation			
SD (mmol/L, M (P_25_, P_75_))	2.50 (1.83, 3.25)	2.76 (2.01, 3.57)	<0.001
CV (%, M (P_25_, P_75_))	28.86 (22.70, 34.83)	29.80 (23.47, 36.13)	0.009
LAGE (mmol/L, M (P_25_, P_75_))	9.30 (6.20, 13.10)	10.10 (7.00, 14.40)	0.001
Other indicators of management			
Nosocomial infection (%)	5.42	8.05	0.034
LOS (M (P_25_, P_75_))	7 (3, 12)	8 (4, 14)	<0.001
Hospital cost (ten thousand, M (P_25_, P_75_))	1.62 (1.01, 3.35)	1.89 (1.29, 4.47)	<0.001

The rows of “Diagnosed diabetes patients” and “Detection rate of hyperglycemia” referred to the proportion within the full cohort of people admitted to the hospital, while other rows referred to the proportion within the hyperglycaemic sub-groups.

BMI: body mass index; SD: standard deviation of blood glucose value; CV: coefficient of variation of blood glucose; LAGE: largest fluctuation range of blood glucose; LOS: length of stay.

Detection rate of hyperglycemia = hyperglycemia / all participants; Incidence of hyperglycemia = the frequency of detected hyperglycemia / total detecting frequency.

### Management indicators related to blood glucose

The team management group had a higher blood glucose detection rate (P <0.01), glycosylated haemoglobin detection rate (P <0.01), and testing frequency per person per day (P <0.01) than the routine consultation group (Table 2).

### Indicators of blood glucose control

Compared to the routine consultation group, the average blood glucose and rates of hyperglycaemia (P <0.01) and critical hyperglycaemia (P<0.01) were significantly lower in the team management group. The incidence of hypoglycaemia (P>0.05) was not significantly different between the routine consultation group and team management group, while the team management group had a lower rate of clinically significant hypoglycaemia (P<0.05) and a higher target blood glucose compliance rate (P<0.01) (Table 2).

### Index of blood glucose fluctuation

The blood glucose drift (P<0.01), blood glucose coefficient of variation (P<0.01) and maximum blood glucose fluctuation range (P<0.01) of the team management group were all lower than those of the routine consultation group (Table 2).

### Other indicators of management effectiveness

The team management group had shorter hospital stays (P<0.01), lower rates of nosocomial infections (P<0.05) and lower hospital costs than the routine consultation group (Table 2).

### Univariate analysis identified some independent variables associated with hospital stay and inpatient diabetes cost

The independent variables associated with hospital stay were average blood glucose level, testing frequency per person per day, blood glucose drift (blood glucose drift, blood glucose coefficient of variation and maximum blood glucose fluctuation range have multicollinearity, so only blood glucose drift was chosen as an independent variable) and the occurrence of nosocomial infection (Table 3). (2) The independent variables associated with hospital cost were average blood glucose level, blood glucose drift, occurrence of nosocomial infection, and length of hospital stay (Table 4). Multiple linear regression analysis showed that blood glucose drift and nosocomial infection occurrence determined the length of hospital stay, and the length of hospital stay was more strongly associated with nosocomial infection occurrence. Hospital cost depended on the length of hospital stay, the average blood glucose level, and the occurrence of hospital infection. Among all these factors, the length of hospital stay was the most important factor for hospital cost (Tables 5 and 6).

**Table 3:** Univariate analysis of hospital days.

	Unstandardized coefficients		Standardized coefficients	*t*	*P* value
	B	Standard error			
Average blood glucose	0.074	0.010	0.176	7.181	<0.001
Frequency of blood glucose testing per person per day	0.056	0.019	0.074	2.967	0.003
Nosocomial infection	1.581	0.071	0.487	22.396	<0.001
CV	0.156	0.017	0.223	9.214	<0.001

CV: coefficient of variation of blood glucose.

**Table 4:** Univariate analysis of hospital cost.

	Unstandardized coefficients		Standardized coefficients	*t*	*P* value
	B	Standard error			
Average blood glucose	0.088	0.013	0.170	6.931	<0.001
Nosocomial infection	1.701	0.091	0.420	18.629	<0.001
CV	0.165	0.021	0.191	7.812	<0.001
The length of hospital stay	0.955	0.020	0.767	48.098	<0.001

CV: coefficient of variation of blood glucose.

**Table 5:** Multiple linear regression analysis of hospital day.

	Unstandardized coefficients		Standardized coefficients	*t*	*P* value
	B	Standard error			
Constant	1.616	0.044		36.637	<0.001
Nosocomial infection	1.532	0.069	0.471	22.108	<0.001
CV	0.128	0.015	0.184	8.647	<0.001

CV: coefficient of variation of blood glucose.

**Table 6:** Multiple linear regression analysis of hospitalization costs.

	Unstandardized coefficients		Standardized coefficients	*t*	*P* value
	B	Standard error			
Constant	7.815	0.083		93.821	<0.001
The length of hospital stay	0.909	0.023	0.730	39.592	<0.001
Nosocomial infection	0.254	0.074	0.063	3.457	0.001
Average blood glucose	0.020	0.008	0.038	2.339	0.019

## Discussion

Hyperglycaemia, a condition that causes serious health problems and poorer short-term prognosis, is fairly common in adults. Previous studies have reported that the incidence of hyperglycaemia was 32% and 29.6% in the United States and China, respectively [11, 12]. Blood glucose levels outside of the normal range are related to a higher incidence of acute and chronic complications, longer LOS, higher risk of mortality and higher hospital cost [12, 13].

For participants whose glucose levels or therapeutic schedule cannot be tested or adjusted in a timely manner by the traditional consultation methods blood glucose data must be manually recorded during hospitalization [11, 14]. Standardized blood glucose monitoring methods to manage diabetes mellitus are urgently needed and can improve the blood glucose compliance rate [15–17]. In the present study, we proposed a new management mode by combining professional endocrinologists with a network blood glucose monitoring system. Compared with the routine consultation group, the team management group had a higher detection rate of hyperglycaemia and glycosylated haemoglobin, which overcame the insufficient detection rate of haemoglobin A1c (HbA1c) in hospitals [18]. In the efficient management group, hyperglycaemia, severe hyperglycaemia and clinically significant hypoglycaemia occurred less often because patients’ blood glucose was managed in a timely manner.

We found that the blood glucose drift, blood glucose coefficient of variation and maximum blood glucose fluctuation of patients were all reduced in the team management group. The awareness of hyperglycaemia prevention increased because blood glucose fluctuations could be observed in a timely manner. Blood glucose fluctuations reflect the unstable state of blood glucose. Larger blood glucose fluctuations indicate a poorer prognosis of hospitalized patients [19, 20]. In the USA, point-of-care testing (POCT) has basically implemented networking techniques, and endocrinologists can check the participants’ blood glucose anytime, allowing for the tracking of fluctuations [21]. In our hospital, blood glucose data from the whole hospital could be effectively integrated after connecting the monitoring system with the management system, ultimately improving detection efficiency.

A previous study showed that a blood glucose management team combined with a blood glucose monitoring network system and endocrinologists could shorten hospitalization time, decrease the readmission rate within 30 days, and ultimately reduce hospitalization costs [22]. In this study, the LOS and hospitalization cost of patients were lower in the team management group than in the routine consultation group. Moreover, bedside education, which is involved in blood glucose management based on network management, can result in better control of participants’ blood glucose levels and reduce hospital costs. In addition, other studies indicated that information system management of participants’ blood glucose reduced the incidence of hypoglycaemia in addition to reducing the blood glucose level of participants [23–25].

This study has several limitations. First, our data were retrospectively collected in a single centre, which might have led to an unavoidable selection bias. Second, the data were collected within two different time intervals (one year apart). Therefore, changes, such as differences in guidelines, availability of medicine, staff, environmental factors and socioeconomic factors, would influence the results.

## Conclusions

This retrospective study revealed that information-based glucose management improved participants’ blood glucose compliance rate, reduced blood sugar fluctuations, reduced the incidence of nosocomial infections, shortened hospital stay and reduced hospital costs. Moreover, diabetes patients significantly improved their understanding of their illness and their self-management effectiveness as a result of the basic diabetes education guided by certified diabetes nurse educators. Above all, the implementation of such a practical computer-assisted consultation model can be of great help for the management of blood glucose.

## Supporting information

S1 Checklist STROBE Statement—Checklist of items that should be included in reports of observational studies. (DOCX)
